# Associations of novel blood-derived markers of inflammation with blood pressure, arterial stiffness and heart rate in young adults

**DOI:** 10.3389/fcvm.2025.1678178

**Published:** 2025-11-25

**Authors:** Richard J. Woodman, Angelo Zinellu, Trevor A. Mori, Lawrie J. Beilin, Arduino A. Mangoni

**Affiliations:** 1 College of Medicine and Public Health, Flinders University, Adelaide, SA, Australia; 2 Department of Biomedical Sciences, University of Sassari, Sassari, Italy; 3 Royal Perth Hospital Unit, Medical School, University of Western Australia, Perth, WA, Australia; 4 Department of Clinical Pharmacology, Flinders Medical Centre, Southern Adelaide Local Health Network, Adelaide, SA, Australia

**Keywords:** blood pressure, arterial stiffness, C-reactive protein, markers of inflammation, platelets, HDL-cholesterol, white blood cell counts

## Abstract

**Background:**

Although C-reactive protein (CRP) is often used to assess inflammation and can predict high blood pressure (BP) and arterial stiffness, novel measures of inflammation derived from platelets, white blood cell counts, and high-density lipoprotein cholesterol (HDL-C) may also indicate possible hypertension and arterial stiffening.

**Methods:**

We assessed the association between CRP and novel inflammatory markers and clinical BP, arterial stiffness, and heart rate (HR) in Gen2 Raine Study participants in Western Australia aged 17–22 years. Arterial stiffness was assessed using pulse-wave velocity (PWV), augmentation index (AIx), and pulse pressure (PP). Inflammatory markers included high-sensitivity CRP (hsCRP); hsCRP-to-albumin ratio; lymphocyte, monocyte, neutrophil, and platelet counts; neutrophil-to-lymphocyte ratio (NLR); platelet-to-lymphocyte ratio (PLR); mean platelet volume; neutrophil percentage-to-albumin ratio; monocyte/HDL-C ratio; neutrophil/HDL-C ratio; Prognostic Nutritional Index and the Systemic Inflammation Index (SII).

**Results:**

Males (*N* = 363) had higher systolic BP (SBP) (*Δ* = 9.1 mmHg, *p* < 0.001), lower diastolic BP (DBP) (*Δ* = −0.9 mmHg, *p* < 0.001), and higher PWV (*Δ* = 0.37 m/sec; *p* < 0.001) than females (*N* = 330). The lymphocyte count, monocyte/HDL-C ratio, and neutrophil/HDL-C ratio were positively associated with SBP, PP, and AIx. The platelet count was positively associated with SBP, DBP, and PP. Most inflammatory indices were associated with HR, and associations with SBP, DBP, AIx, and PWV were stronger in males than in females. The hsCRP and SII levels were not associated with SBP, DBP, PP, AIx, or PWV.

**Conclusions:**

The lymphocyte count, monocyte/HDL-C ratio, neutrophil/HDL-C ratio, and platelet count showed more robust associations with blood pressure and arterial stiffness in young adults compared to hsCRP and SII, suggesting their potential utility in this specific context.

## 1 Introduction

Systemic inflammation is closely associated with vascular alterations, including endothelial dysfunction and increased arterial stiffness (1) and traditional markers of inflammation, such as C-reactive protein (CRP), have been associated with hypertension in large-scale studies (2, 3). In older adults, low-grade inflammation is associated with cardiometabolic multimorbidity (4) In healthy younger adults, inflammatory markers, including cytokines, have been associated with increased blood pressure (BP) over 4.5 years (5), demonstrating that excess inflammation can exert significant negative effects on BP at an early age. The identification of easily attainable inflammatory markers for predicting BP elevation at a young age would facilitate more targeted monitoring and interventions to prevent future hypertension and cardiovascular disease. For example, a modified intake of dietary fatty acids, salt, and ultra-processed foods, which are associated with increased inflammation and BP (6–8), might reduce the incidence of hypertension in young adults.

Although inflammation predicts some forms of hypertension and its development, there is no consensus on the best marker for this purpose (9). Most studies addressing the relationship between inflammation and BP have measured CRP as a non-specific marker of inflammation (2, 10) but many additional inflammatory markers, including neutrophil, lymphocyte, monocyte, and platelet counts, can be derived from routine hematological blood tests. Others include the neutrophil-lymphocyte ratio (11), lymphocyte-monocyte ratio (12), platelet-lymphocyte ratio (12), mean platelet volume (13) and the systemic inflammation index (SII) (14). Albumin and HDL-cholesterol (HDL-C) have also been used to normalize some of these counts to provide additional potential markers, including the monocyte-to-HDL-C ratio (15), neutrophil-to-HDL-C ratio (16), CRP-to-albumin ratio (17) and neutrophil percentage-to-albumin ratio (18). WBC count may also be a more specific marker of hypertension-related inflammation, since T-lymphocytes appear essential for the development of hypertension, and the central nervous system contributes to hypertension via mechanisms such as peripheral T-lymphocyte activation and vascular inflammation (1, 19). T lymphocytes and vascular inflammation also contribute to stress-dependent hypertension (1). Elevated monocyte-to-HDL and neutrophil-to-HDL ratios suggest a higher inflammatory state relative to protective HDL-C, and are associated with an increased risk of cardiovascular events (16, 20). CRP-to-albumin (17) and neutrophil-percent-to-albumin (18) ratios combine an inflammatory marker with a measure of nutritional status and have been studied in cardiovascular and kidney diseases. However, there is relatively little information regarding whether these indices can better predict BP elevation than CRP level in young adults.

**Figure 1:**
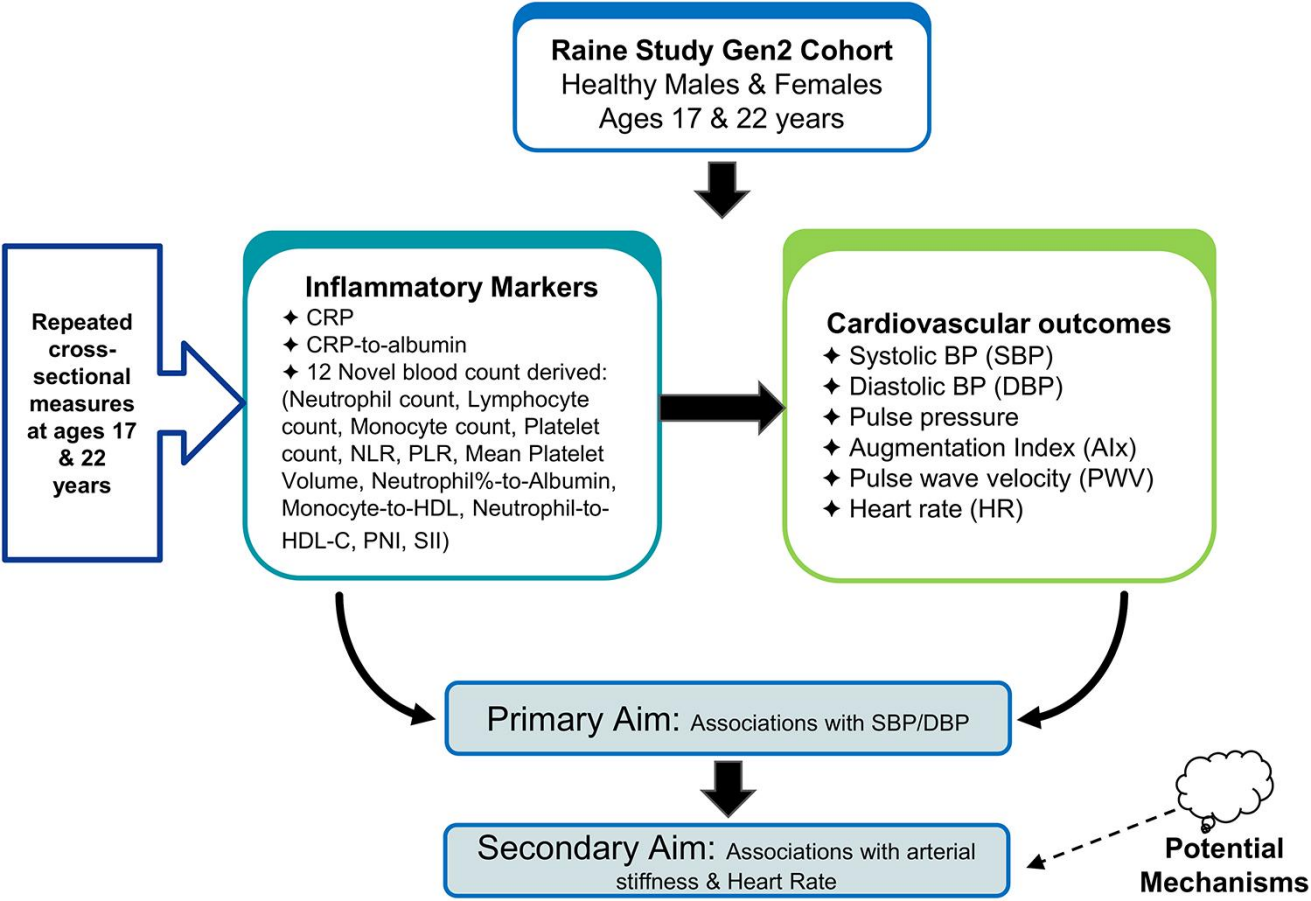
Study design and aims flowchart: primary aims of the study, investigating the associations between markers and cardiovascular outcomes in the raine study Gen2 cohort.

The aims of the study and study design are described in the flow diagram in Figure 1. Our primary aim was to investigate the association between CRP and CRP-to-albumin and 12 novel markers of inflammation (Neutrophil count, Lymphocyte count, Monocyte count, Platelet count, Neutrophil-to-lymphocyte ratio, Platelet-to-lymphocyte ratio, Monocyte-to-platelet volume, SII, monocyte-to-HDL ration, Neutrophil-to-HDL ratio, neutrophil percent-to-albumin and PNI), and systolic BP (SBP) and diastolic BP (DBP)). Our secondary aim was to investigate whether these blood count-derived inflammatory indices and CRP levels were associated with direct and indirect measures of arterial stiffness (pulse pressure (PP), augmentation index (AIx), pulse wave velocity (PWV), and heart rate (HR)) to assess potential mechanisms. We obtained repeat cross-sectional measures of blood counts and clinic BP at ages 17 and 22 years and AIx and PWV obtained at age 17 in a population of healthy males and females from the Raine Study.

## 2 Materials and methods

### 2.1 Participants

The Raine Study (https://www.rainestudy.org.au) represents a large cohort of Western Australian offspring studied from 18 weeks of gestation to ascertain the relative contributions of familial risk factors, fetal growth, placental development, and environmental insults to outcomes in infancy and to the precursors of adult morbidity. A total of 2,900 women (Gen1) were enrolled in this study from to 1989–1991. Thereof 2,868 live births (Gen2) were prospectively followed up at regular intervals from birth, and demographic, lifestyle, clinical, and biochemical information was collected through questionnaires and clinical assessments (21). The Human Ethics Committees at King Edward Memorial Hospital, Princess Margaret Hospital for Children, and the University of Western Australia in Perth approved all the recalls of the cohort. This study included 692 Gen2 participants with available clinical BP data and inflammatory markers at both 17 and 22 years of age. The selection process is described in further detail in the CONSORT flowchart (Supplementary Figure S1).

### 2.2 Demographic and clinical data

Demographic data, including smoking status (yes/no), level of physical activity (low/moderate/high), use of oral or hormonal contraceptives (at ages 17 and 22), and alcohol consumption by age 17 (none/just-a-few-sips/less-than-10-sips/more-than-10-sips), were recorded in the medical questionnaires. An international physical activity questionnaire (IPAQ) recorded the metabolic equivalents (METs) at 17 and 22 years of age. Clinical assessments included the waist-to-hip ratio (WHR). Information on the family history of high BP in either the mother or father was recorded at the age of eight years. Supplementary Figure S1 provides details on the number of individuals assessed for each measurement at 17- and 22-years, and the extent of missing information.

### 2.3 Clinic BP and heart rate

Resting clinic BP and HR were measured after overnight fasting with an appropriate cuff size for arm circumference, using a semi-automated oscillometric monitor (DINAMAP ProCare 100 vital signs monitor; GE Healthcare, USA). Recordings were obtained from the right arm after the participant had been seated for ≥5 min. Six measurements were taken 2 min apart and the first recording was removed from the analysis (22) before averaging the remaining five readings.

### 2.4 Arterial stiffness

The measures of arterial stiffness included PWV, AIx, and PP, which were calculated as the mean systolic BP minus the mean diastolic BP. AIx was measured using pulse wave analysis (PWA) (Sphygmocor, software version 1.2, AtCor Medical Pty Ltd, Sydney, Australia). PWA data were collected from a supported radial artery with the wrist facing upward and data were captured after a consistent waveform was maintained for 10 s. The test was repeated until at least two captures were recorded with a quality index of >80. AIx was defined as the difference in the second and first systolic pressure peaks as a percentage of pulse pressure and was corrected to a heart rate of 75 bpm using AIx@ HR75 = [−0.48 × (75−HR)] + AIx (23). For PWV measurements, three ECG leads were attached to the left, right, and left arms. Tonometers were applied to the carotid artery and distal dorsalis pedis, and the distance (mm) between the manubrium sternum and the two sampling sites was measured. PWV was calculated by dividing the distance between the tonometers by the transit time of the arterial pulse wave (24). The day-to-day coefficient of variation for the PWV was 5%.

### 2.5 Inflammatory markers

The 14 inflammatory markers studied included high-sensitivity CRP, CRP/Albumin, Lymphocytes, Monocytes, Neutrophils, Platelets, Neutrophil-to-Lymphocyte Ratio, Platelet-to-Lymphocyte Ratio, Monocyte-to-Platelet Volume, Neutrophil-percent/Albumin ratio, Monocyte/HDL-C ratio, Neutrophil/HDL-C ratio, the Prognostic Nutritional Index (PNI) and the Systemic Inflammation Index (SII) calculated as [platelet count × neutrophil count]/lymphocyte count (25). The PNI was assessed using the following formula: [5 × lymphocyte count (10^9^/L)] + serum albumin (g/L). Serum hsCRP, stored at −80°C within 2 h of collection, was analyzed using an immunoturbidimetric method on an Architect c16000 Analyzer (Abbott Core Laboratory, Illinois, United States). A single aliquot of whole blood was processed for full blood count, including circulating monocytes, neutrophils, lymphocytes, and platelets. HDL-C level was determined in heparin–manganese supernatants (26). High-sensitivity CRP (hsCRP), HDL-C, and blood counts were analyzed by PathWest Laboratory (Royal Perth Hospital) (27).

### 2.6 Dietary intake and alcohol consumption

Information on dietary intake, which was used in regression adjustment, was estimated using the 74-item semiquantitative Dietary Questionnaire for Epidemiological Studies (DQESV2) FFQ developed by the Cancer Council of Victoria, Australia (28). Standardized scores for Healthy and Western diets at the age of 17 years were calculated using factor analysis of major food group intake obtained from a semi-quantitative dietary recall questionnaire designed by Australia's Commonwealth Scientific and Industrial Research Organization (CSIRO) (29, 30). These two extracted factors explained 13% (Western dietary pattern) and 8.5% (Healthy dietary pattern) of the total variance in food intake (31). Additional information from the CSIRO dietary food recall records included sodium and fiber intake, frequency of fruit and vegetable consumption, and the standard number of alcoholic drinks consumed per week, captured from a medical questionnaire at age 20. This was recorded both as grams of alcohol per day for a typical week and using three categories (<1 standard drink/day, 1–3 standard drinks/day, and >3 standard drinks/day).

### 2.7 Statistical analysis

The study population was described using the mean ± SD for normally distributed variables, median (interquartile range) for non-normally distributed variables, and frequencies (percentages) for categorical variables. Differences between males and females were assessed using an independent *t*-test or Mann–Whitney *U*-test for continuous variables and a chi-squared test for frequency data. Associations between inflammatory markers and clinical systolic BP, diastolic BP, PP, and HR were assessed using a linear mixed-effects model with subject ID included as a random intercept and an unstructured covariance matrix for the residuals. Random effects accounted for repeated measures on the same individual at 17 and 22 years of age. The associations between inflammatory markers and AIx and PWV (measured at 17 years of age) were assessed using a generalized linear model with Gaussian distribution and an identity link. The exposure of interest for each model was one of the 14 individual inflammatory markers included as a continuous variable without transformation. The dependent variables were the mean clinical SBP, DBP, PP, HR, AIx, and PWV. Associations were determined with and without adjustment for potential confounders, including age, waist-to-hip ratio, family history of hypertension, healthy and Western diet factor analysis scores, frequency of fruit and vegetable consumption, alcohol intake (g/day), sodium and fiber intake, METs per week, and physical activity category (0/1/2). All regression model results were reported as the mean change (β) (95% confidence interval) for a one standard deviation increase in each inflammatory marker. Owing to missing data for some of the covariates (Supplementary Figure S1), multiple imputation with chained equations (MICE) was used to create 20 multiply imputed datasets that were used for all analyses. The MICE routine uses predictive mean matching to impute categorical variables, and linear regression to impute continuous covariates. The non-missing variables included in the imputation were systolic and diastolic BP measurements, participant ID, and visits (17 vs. 22 years). All analyses were performed using Stata (version 17.0; StataCorp, U.S.A.).

## 3 Results

### 3.1 Study population

Of the original 2,868 Gen2 participants, 693 had complete data on inflammatory markers and clinic BP at ages 17 and 22 years. The CONSORT flow diagram (Supplementary Figure S1) describes the inclusion and exclusion of participants from the original cohort.

Table 1 shows the demographic and lifestyle characteristics of 693 participants. There were slightly more males (*n* = 363, 52.4%) than females (*n* = 330, 47.6%), and the overall mean (±SD) WHR across all participants was 0.81 ± 0.07 at age 17 and 0.83 ± 0.07 at age 22, with females having a lower WHR than males at age 17 and 22 (*p* < 0.001). Participants generally met fruit, vegetable, and alcohol recommendations at ages 17 and 22 years, according to the Australian Guide to Healthy Eating (AGHE). Western diet factor scores and healthy diet factor scores at age 17 were comparable to the full Raine Study Gen2 cohort with means (±SD) of −0.05 ± 0.82 and 0.05 ± 0.89 respectively and close to zero. However, females had a slightly healthier profile than males for the Western diet factor score (−0.35 ± 0.70 vs. 0.25 ± 0.83, *p* < 0.001) but were similar for the healthy diet factor score (0.12 ± 0.90 vs. −0.03 ± 0.89, *p* = 0.076). The median alcohol consumption at age 20 was slightly lower among females than males [7.1 (0.0–17.1) g/day vs. 11.4 (0.0–30.0) g/day, *p* < 0.001]. Most participants (*N* = 547, 84.8%) were non-smokers at the age of 22 years. Males were higher in the physical activity category and in mean MET mins/week at both ages 17 and 22 than females. Table 2 describes the inflammatory markers BP, HR, AIx, and PWV in the participants. Most of the study participants were normotensive (SBP < 130 mmHg and DBP < 80 mmHg) (32) at age 17 (94.4%) and at age 22 (83.6%), with the overall mean (±SD) SBP at age 17 being 117.3 ± 9.5 for males and 108.6 ± 9.3 mmHg for females (*p* < 0.001). At age 22, the corresponding mean SBP was 123.3 ± 10.6 and 113.7­ ± 9.3 mmHg (*p* < 0.001). Among the inflammatory markers, monocyte count (*p* = 0.009 and *p* < 0.001) and monocyte/HDL-C ratio (*p* < 0.001 for each) were higher in males than in females at ages 17 and 22, while hsCRP was higher in females than in males at both ages (*p* < 0.001 for each), as was the platelet count (*p* < 0.001 for each).

**Table 1:** Demographics, diet, and lifestyle characteristics of Gen2 participants (*N* = 693) at 17- and 22-years of age.

	All individuals	Males	Females	*P*-value^a^
	*N* = 693	*N* = 363	*N* = 330	
	(100%)	(52.4%)	(47.6%)	
Age, years (mean ± SD)				
At age 17	17.02 ± 0.23	17.01 ± 0.22	17.03 ± 0.25	0.293
At age 22	22.10 ± 0.59	22.14 ± 0.59	22.07 ± 0.59	0.146
Waist-Hip-Ratio, mean(±SD)				
At age 20	0.81 ± 0.07	0.83 ± 0.06	0.78 ± 0.07	<0.001
At age 22	0.83 ± 0.07	0.86 ± 0.06	0.79 ± 0.07	<0.001
Family history of High blood pressure^b^, *n* (%)				
No	477 (87.2)	252 (87.2)	225 (87.2)	0.997
Yes	70 (12.8)	37 (12.8)	33 (12.8)	
Fruit consumption frequency (at age 17), *n* (%)				
0: Rarely or never	14 (2.0)	11 (3.0)	3 (0.9)	0.052
1: 1–2 times/month	33 (4.8)	22 (6.1)	11 (3.3)	
2: 1–2 times/week	125 (18.0)	68 (18.7)	57 (17.3)	
3: 3–5 times/week	248 (35.8)	123 (33.9)	125 (37.9)	
4: 6 + times/week	258 (37.2)	128 (35.3)	130 (39.4)	
Missing	15 (2.2)	11 (3.0)	4 (1.2)	
Fruit consumption category (at age 17)^c^				
Median (IQR)	3 (2–4)	3 (2–4)	3 (3–4)	0.064
Mean ± SD	3.04 ± 0.97	2.95 ± 1.04	3.12 ± 0.88	0.018
Vegetable consumption frequency (at age 17), *n* (%)				
0: Rarely or never	3 (0.4)	2 (0.55)	1 (0.3)	0.125
1: 1–2 times/month	11 (1.6)	6 (1.65)	5 (1.5)	
2: 1–2 times/week	68 (9.8)	39 (10.7)	29 (8.8)	
3: 3–5 times/week	229 (33.0)	128 (35.3)	101 (30.6)	
4: 6 + times/week	368 (53.1)	177 (48.8)	191 (57.9)	
Missing	14 (2.0)	11 (3.0)	3 (0.9)	
Vegetable consumption category (at age 17)^c^				
Median (IQR)	4 (3–4)	4 (3–4)	4 (3–4)	0.035
Mean (SD)	3.40 ± 0.77	3.34 ± 0.79	3.46 ± 0.75	0.052
Sodium intake^c^ (at age 17), mg/day (mean ± SD)	3,023 ± 1,296	3,510 ± 1,364	2,541 ± 1,017	<0.001
Dietary factor analysis scores^c^ (mean ± SD)				
Western diet score (age 17)	−0.05 ± 0.82	0.25 ± 0.83	−0.35 ± 0.70	<0.001
Healthy diet score (age 17)	0.05 ± 0.89	−0.03 ± 0.89	0.12 ± 0.90	0.076
Weekly alcohol consumption (age20)^d^				
Median (IQR) g/ day	8.6 (0.0–22.9)	11.4 (0.0–30.0)	7.1 (0.0–17.1)	<0.001
Alcohol categories (age 20)^d^				
Less than 1 standard drink	502 (88.1)	243 (86.8)	259 (89.3)	
1–3 standard drinks	45 (7.9)	23 (8.2)	22 (7.6)	
More than 3 standard drinks	23 (4.0)	14 (5.0)	9 (3.1)	0.486
Ever drunk (age 17)				
No	40 (5.9)	25 (7.1)	15 (4.6)	
Just a few sips	61 (9.0)	32 (9.1)	29 (8.9)	
Yes, fewer than 10 sips	97 (14.3)	43 (12.2)	54 (16.6)	
Yes, more than 10 sips	481 (70.8)	253 (71.7)	228 (69.9)	0.248
Smoking status (age 22) *n* (%)				
No	547 (84.8)	278 (84.0)	269 (85.7)	
Yes	98 (15.2)	53 (16.0)	45 (14.3)	0.552
Physical activity category (age 17), *n* (%)				
Low	72 (10.6)	28 (7.9)	44 (13.4)	
Moderate	275 (40.3)	120 (34.0)	155 (47.1)	
High	335 (49.1)	205 (58.1)	130 (39.5)	<0.001
Physical activity category (age 22), *n* (%)				
Low	114 (16.7)	45 (12.75)	69 (21.0)	
Moderate	193 (28.3)	79 (22.4)	114 (34.65)	
High	375 (55.0)	229 (64.9)	146 (44.4)	<0.001
MET minutes/week, mean (SD)				
Age 17	1,421 ± 2,994	1,702 ± 3,480	1,127 ± 2,353	0.018
Age 22	5,429 ± 4,000	6,257 ± 4,174	4,383 ± 3,513	<0.001
Oral contraceptive use (age 22), *n* (%)				
Age 17	109 (33.4)	N/A	109 (33.4)	
Age 22	158 (49.8)	N/A	158 (49.8)	N/A

a *P*-value for differences between sexes.

b Family history refers to either the mother or the father. ^c^Data for the specified waves were obtained using dietary and medical questionnaires.

d One standard drink = 10 g alcohol.

**Table 2:** Blood pressure, hypertension status (stage 1/stage 2), heart rate, augmentation index, pulse-wave velocity, and inflammatory markers of participants at 17- and 22-years (*N* = 693).

Clinical characteristic	All individuals	Males	Females	*P*-value^a^
	*N* = 693	*N* = 363	*N* = 330	
	(100%)	(52.4%)	(47.6%)	
Systolic BP^b^, mmHg				
At age 17	113.1 ± 10.4	117.3 ± 9.5	108.6 ± 9.3	<0.001
At age 22	118.7 ± 11.1	123.3 ± 10.6	113.7 ± 9.3	<0.001
Diastolic BP^b^, mmHg				
At age 17	58.6 ± 6.3	57.9 ± 6.5	59.4 ± 6.1	0.002
At age 22	67.2 ± 7.1	67.0 ± 7.3	67.3 ± 7.1	0.525
Normotensive BP, *n* (%)				
At age 17	654 (94.4)	332 (91.5)	268 (73.8)	<0.001
At age 22	579 (83.6)	322 (97.6)	311 (94.2)	<0.001
Stage 1 hypertension^c^				
At age 17	29 (4.2)	22 (6.1)	7 (2.1)	0.012
At age 22	97 (14.0)	77 (21.2)	20 (6.1)	<0.001
Stage 2 hypertension^c^				
At age 17	8 (1.15)	7 (1.9)	1 (0.3)	0.071
At age 22	23 (3.3)	19 (5.2)	4 (1.2)	0.005
HR, bpm^b^				
At age 17	65.4 ± 9.9	63.5 ± 10.0	67.5 ± 9.3	<0.001
At age 22	75.5 ± 11.2	72.8 ± 11.4	76.3 ± 10.7	<0.001
AIx (Age 17), mmHg	(*N* = 645)	(*N* = 344)	(*N* = 301)	0.062
At age 17	−1.57 ± 10.14	−2.27 ± 11.0	−0.78 ± 9.07	
PWV (Age 17), m/sec	(*N* = 666)	(*N* = 349)	(*N* = 317)	<0.001
At age 17	6.45 ± 0.73	6.62 ± 0.74	6.25 ± 0.67	
Inflammatory markers (Age 17), median (IQR)				
Monocyte count (per mL)	0.55 (0.45, 0.67)	0.57 (0.47, 0.69)	0.54 (0.43, 0.65)	0.009
Lymphocyte count (x10^9^/L)	2.75 (2.36, 3.32)	2.73 (2.31, 3.19)	2.44 (2.82, 3.44)	0.018
Neutrophil count (x10^9^/L)	3.15 (2.53, 3.88)	3.05 (2.47, 3.68)	3.27 (2.68, 4.14)	<0.001
Platelet count (x10^9^/L)	270 (235, 307)	260 (226,287)	281 (247,327)	<0.001
Platelet-Lymphocyte ratio	97 (79, 119)	94 (78, 114)	98 (80, 121)	0.062
Neutrophil-Lymphocyte ratio	1.13 (0.90, 1.42)	1.13 (0.92, 1.35)	1.13 (0.89, 1.52)	0.190
Systemic Inflammation Index	302 (231, 397)	289 (216, 363)	242 (331, 442)	<0.001
hsC-reactive protein	0.51 (0.20, 1.32)	0.43 (0.16, 0.97)	0.67 (0.25, 2.02)	<0.001
hsCRP/albumin	0.011 (0.00,0.03)	0.01 (0.00,0.02)	0.02 (0.01,0.05)	<0.001
Mean platelet volume (femtolitres)	8 (8, 9)	8 (8, 9)	8 (8, 9)	0.827
Neutrophils/HDL-C	2.48 (1.90,3.26)	2.52 (1.95,3.20)	2.44 (1.79, 3.28)	0.210
Monocytes/HDL-C	0.44 (0.38, 0.57)	0.49 (0.38, 0.61)	0.39 (0.30, 0.50)	<0.001
Neutrophil percent/albumin	1.01 (0.88, 1.15)	0.98 (0.85, 1.08)	1.04 (0.90, 1.22)	<0.001
PNI	460 (440, 481)	470 (454, 490)	447 (432, 470)	<0.001
Inflammatory markers (Age 22), median (IQR)				
Monocytes	0.58 (0.47,0.70)	0.61 (0.51, 0.73)	0.55 (0.45, 0.66)	<0.001
Lymphocytes	2.7 (2.22, 3.17)	2.46 (2.09, 2.93)	2.88 (2.44, 3.40)	<0.001
Neutrophils	3.45 (2.83, 4.31)	3.4 (2.9, 4.2)	3.5 (2.8, 4.4)	0.642
Platelets	220 (194, 252)	210 (189, 235)	233 (206, 271)	<0.001
Platelet-Lymphocyte ratio	83 (69, 102)	84 (71, 102)	81 (68, 101)	0.174
Neutrophil-Lymphocyte ratio	1.30 (1.02, 1.66)	1.19 (0.93, 1.60)	1.35 (1.11, 1.73)	<0.001
Systemic Inflammation Index	285 (218, 382)	286 (229, 383)	284 (201, 381)	0.533
hsC-reactive protein	0.93 (0.41, 2.24)	0.71 (0.35, 1.46)	1.45 (0.50, 3.42)	<0.001
hsCRP/albumin	0.02 (0.01,0.05)	0.02 (0.01, 0.03)	0.03 (0.01, 0.08)	<0.001
Mean platelet volume (femtoliters)	9 (8, 10)	9 (8, 10)	9 (8, 10)	<0.001
Neutrophils/HDL-C	2.68 (1.96, 3.53)	2.94 (2.16, 3.63)	2.31 (1.74, 3.23)	0.642
Monocytes/HDL-C	0.44 (0.34, 0.58)	0.51 (0.40, 0.63)	0.38 (0.29, 0.48)	<0.001
Neutrophil percent/albumin	1.14 (1.02, 1.28)	1.13 (1.02, 1.25)	1.16 (1.02, 1.32)	0.174
PNI	430 (410, 450)	447 (430, 460)	410 (400, 430)	<0.001

BP, blood pressure; HR, heart rate; PWV, pulse wave velocity; Aix, augmentation index; HDL-C, high-density lipoprotein cholesterol; hsCRP, high-sensitivity C-reactive protein. PNI, prognostic nutritional index.

a Mean of five supine clinic blood pressure readings taken after minutes 2, 4, 6, 8, and 10.

b *P*-value for the difference between sexes, IQR, interquartile range.

c Stage 1 hypertension was defined as SBP ≥ 130 and ≤139 mmHg or DBP ≥ 80 and ≤89 mmHg. Stage 2 hypertension was defined as an SBP ≥ of 140 mmHg or DBP ≥ of 90 mmHg (32).

### 3.2 Inflammatory marker correlations

Supplementary Table S1 describes the Spearman correlations between the 14 inflammatory markers and covariates used for adjustment. Several variables, including sex, WHR, Western diet score, MET min/week, and smoking status, were consistently associated with inflammatory markers, including monocytes vs. sex (*ρ* = −0.54, *p* < 0.001), indicating a higher level of monocytes in males than in females. Monocytes/HDL-C were associated with sex (*ρ* = −0.35) and WHR (*ρ* = 0.33), and neutrophils/HDL-C were associated with WHR (*ρ* = 0.28) (*p* < 0.05). Supplementary Figures S2–S8 display scatter plots for the inflammatory markers vs. SBP (Supplementary Figure S2), age (Supplementary Figure S3), sex (Supplementary Figure S4), WHR (Supplementary Figure S5), MET min/week (Supplementary Figure S6), western diet score (Supplementary Figure S7), and smoking (Supplementary Figure S8), respectively.

### 3.3 Regression analysis of inflammatory markers

**Figure 2:**
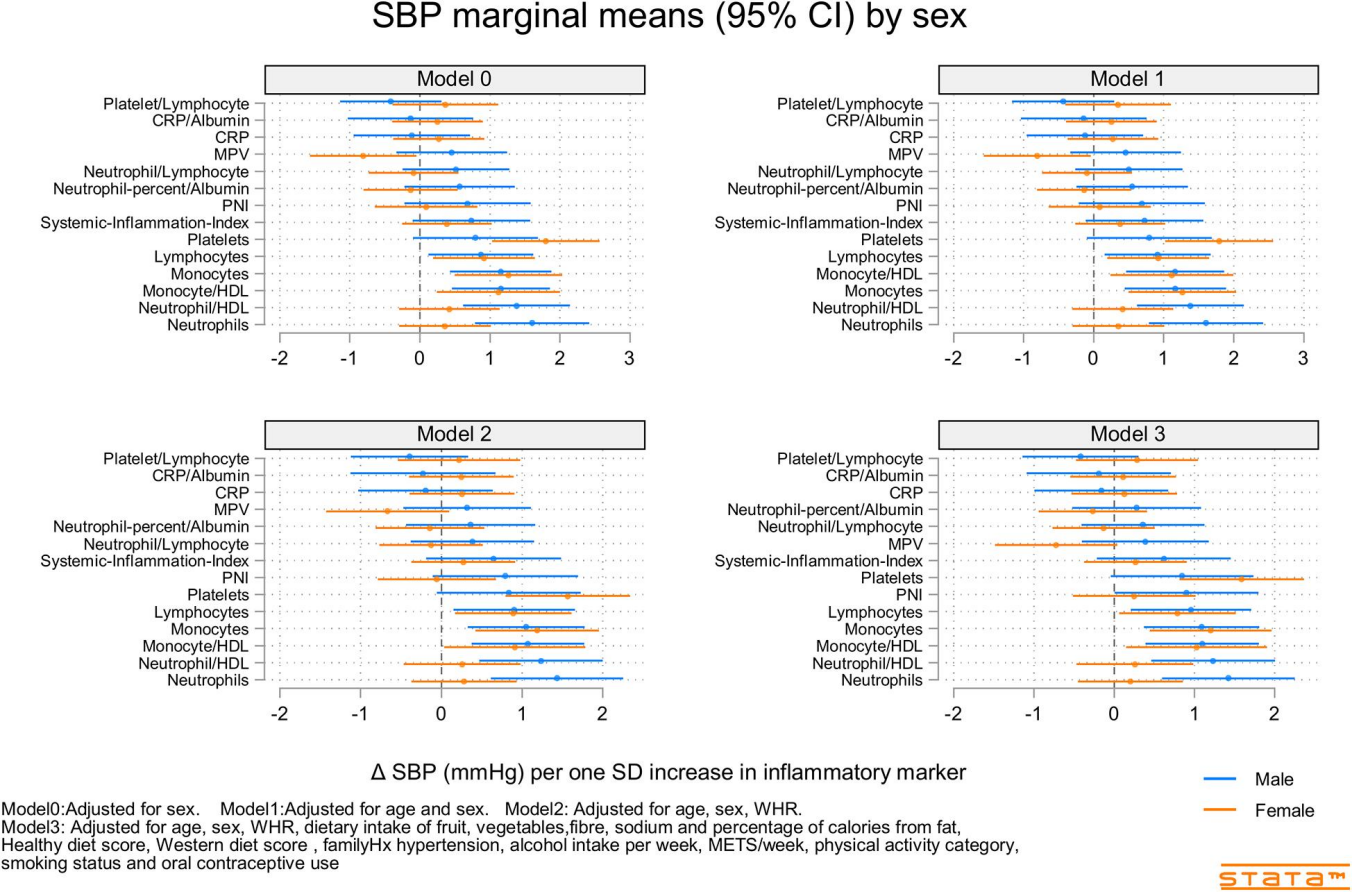
Predicted marginal mean effect of inflammatory markers on clinic systolic blood pressure for males (*N* = 363) and females (*N* = 330).

**Figure 3:**
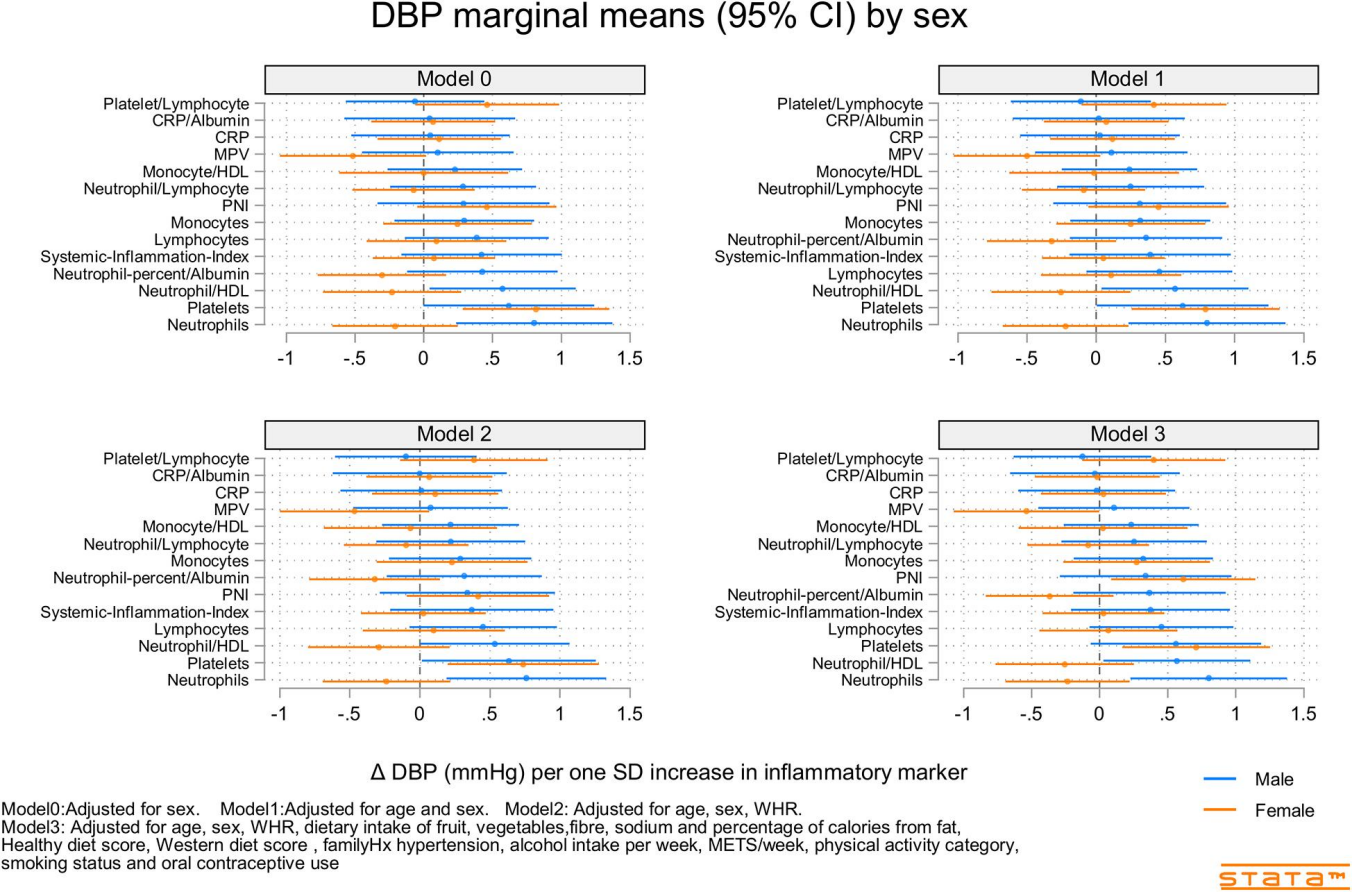
Predicted marginal mean effect of inflammatory markers on clinic diastolic blood pressure for males (*N* = 363) and females (*N* = 330).

**Figure 4:**
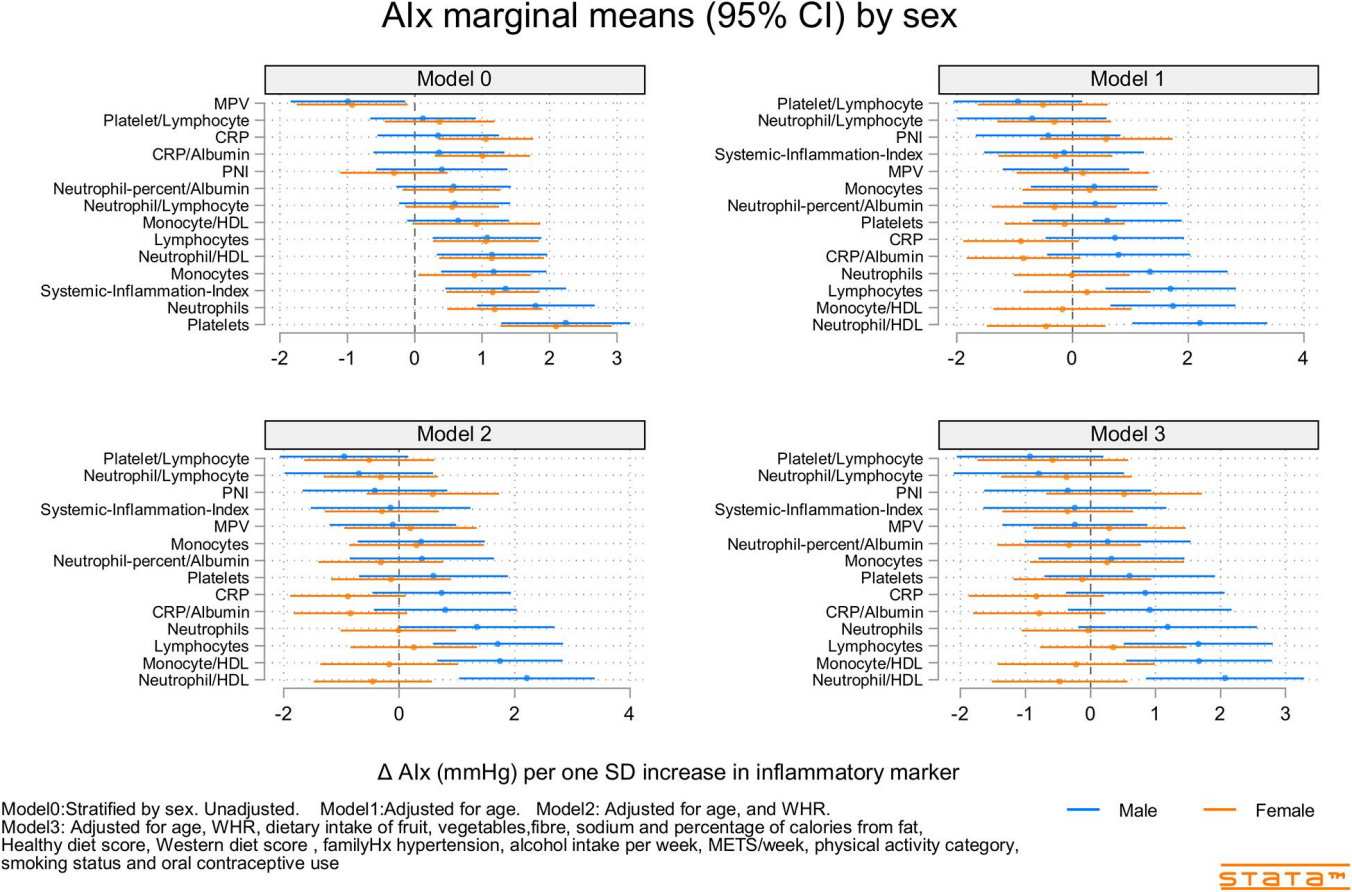
Predicted marginal mean effects of inflammatory markers on AIx for males (*N* = 344) and females (*N* = 301).

Figures 2–4 and Supplementary Figures S9–S11 show the strength of the unadjusted and fully adjusted associations between each inflammatory marker and SBP, DBP, AIx, HR, PP, and PWV, respectively. Supplementary Tables S3, S4 provide details of the fully adjusted associations (Model 3) and the results of testing for effect modification between males and females. SBP was associated with the lymphocyte count, monocyte-to-HDL-C ratio, neutrophil-to-HDL-C ratio, monocyte count, neutrophil count, platelet count, and PNI. DBP was associated with monocyte-to-platelet volume ratio, neutrophil-to-HDL-C ratio, neutrophil count, platelet count, and PNI. PP was associated with lymphocyte, monocyte-to-HDL-C, neutrophil-to-HDL, monocyte, and platelet counts]. AIx was associated with the lymphocyte count, monocyte-to-HDL ratio, and neutrophil-to-HDL ratio. PWV was associated with platelet/lymphocyte ratio.

The most consistently associated inflammatory markers were the lymphocyte count, monocyte-to-HDL-C ratio, and neutrophil-to-HDL-C ratio, which were significantly associated with SBP, PP, and AIx. The platelet count was significantly associated with SBP, DBP, and PP. Among the 14 inflammatory markers, only the hsCRP level, hsCRP-to-albumin ratio, neutrophil-to-lymphocyte ratio, neutrophil percent-to-albumin ratio, platelet-to-lymphocyte ratio, and SII were not associated with SBP, DBP, PP, AIx, or PWV. Heart rate was associated with nine of the 14 inflammatory indices. The monocyte-to-HDL-C, neutrophil-to-lymphocyte, neutrophil-to-albumin, and platelet-to-lymphocyte ratios and PNI were not associated with HR.

### 3.4 Inflammatory marker X sex interactions

Associations between inflammatory markers, BP, and stiffness were consistently stronger in males compared than in females (Supplementary Table S2). Significant interactions included those for SBP (sex X mean platelet volume and Neutrophils), DBP (sex X Neutrophil-to-HDL-C, Neutrophils, and Neutrophil percent-to-albumin), PP (sex × PNI), AIx (sex X Neutrophil-to-HDL-C, Monocyte-to-HDL-C, hsCRP, and hsCRP-to-albumin), and PWV (sex X Lymphocyte count and Neutrophil count).

## 4 Discussion

Within a young and healthy population, our study determined significant associations between novel markers of inflammation derived from routinely measured hematological cell types, including lymphocyte count, monocyte-to-HDL-C ratio, neutrophil-to-HDL-C ratio, BP, and arterial stiffness (AIx and PP). Our study also provided indicators of the potential mechanisms underlying these associations. First, almost all measures of inflammation were significantly associated with heart rate, suggesting that an increased sympathetic tone may contribute. Second, the positive associations between inflammatory markers and measures of arterial stiffness confirm the latter as a potential mediator of increased BP in young adults (33, 34). Third, the stronger associations observed in males than in females may reflect the higher BP and BP variability in males or true differences arising as a consequence of the many different biological functions affected by sex, including immune cell function (35) and inflammatory disease (36).

Although previous studies have observed associations between inflammation, BP, and arterial stiffness (1, 3, 4), this study provides further evidence of these associations in a young and healthy population, and includes novel inflammatory markers. In contrast, CRP was not associated with any measure of BP or stiffness, despite the latter being a predictor of future hypertension in a healthy middle-aged population (2). If the observed BP-inflammatory marker associations reported here reflect the true presence of underlying low-level inflammation and subsequent vascular damage, WBC-derived markers may be more strongly associated with blood pressure and arterial stiffness than CRP in relation to hypertension in young adults. The noticeable increase in BP between the ages of 17 and 22, especially among males, supports the notion that the observed associations might reflect underlying inflammation and vascular damage related to hypertension within a younger population displaying subtle and gradually increasing systemic low-level inflammation (8). In addition, whilst CRP is a general acute-phase reactant reflecting systemic inflammation, cell-derived indices such as lymphocyte count, monocyte/HDL-C ratio, and neutrophil/HDL-C ratio provide insights into specific immune cell populations and their interaction with lipid metabolism. Monocytes, for instance, play a critical role in early atherosclerosis and vascular damage, and their ratio to HDL-C might capture a more nuanced pro-inflammatory state that is strongly associated with endothelial dysfunction, arterial stiffness, and the overall burden of atherosclerotic disease, making it particularly relevant to vascular health in young individuals (15, 37). Similarly, neutrophil activity is implicated in endothelial dysfunction.

The lack of association with some broader inflammatory indices (e.g., NLR, PLR, SII) in our healthy, predominantly normotensive young cohort, in contrast to their utility in older or diseased populations, suggests that the mechanisms linking inflammation to early cardiovascular changes may involve more specific immune cell pathways that are captured by the associated markers in this study. The SII is calculated as the product of platelet count and NLR, and has been proposed as an inflammatory marker to evaluate the prognosis of hepatocellular carcinoma (38) and in patients with CVD (39). NLR has also been used as a marker of systemic inflammation and stress in critically ill patients (40, 41) and PLR as a marker in patients with solid tumors (41). The lack of associations in this study may reflect the different populations involved in the original studies, with our healthy and mostly normotensive populations differing in both underlying levels of inflammation and disease.

Our findings of an association between lymphocytes, monocytes/HDL-C, and neutrophils/HDL-C with arterial stiffness (AIx and PP) support previous studies demonstrating an association between WBCs, vascular function, and arterial stiffness (42, 43). Inflammation is associated with arterial stiffness and impaired vascular function in older adults (1) and increased sympathetic activity may increase and activate T lymphocytes (1, 19). Our observed associations among inflammatory markers, BP, arterial stiffness, and HR support both increased sympathetic activity and arterial stiffening as mechanisms that cause increased BP in a young, healthy population.

Our study has several strengths. We measured and adjusted for a wide range of potential confounders, including important aspects of diet that can influence BP (sodium, fiber, and diet quality), physical activity, WHR, alcohol intake, smoking status, and family history of hypertension. We also used multiple imputations to ensure the inclusion of all subjects in the adjusted models. We measured BP and inflammatory markers at two time points, which reduced the potential for residual error, and considered a wide range of potential markers of inflammation, enabling a comprehensive assessment of the best candidates for a simple surrogate measure of hypertension-related inflammation. Finally, our BP, HR, AIx, and PWV outcomes were aggregated from multiple recordings at each visit, which should reduce the potential for measurement errors and residual confounding.

Our study has several limitations. Although we adjusted for many potential confounders, some of the dietary information used in the adjustment process was obtained at age 20, rather than at ages 17 or 22, when the BP and inflammatory data were obtained. However, since the diets of the Raine Study Gen2 adolescents have been tracked closely into adulthood (29), adjusting for these measures using age 20 data should still be reduced rather than increase the potential for bias. Additionally, we could not assign causality to our findings due to the observational nature of our study. It is possible that inflammatory measures are related to other factors that influence BP, HR, and arterial stiffness, such as physical activity and diet. However, while sex, WHR, Western dietary score, and METs were consistently associated with many inflammatory markers, the observed inflammatory marker-BP associations remained significant after adjusting for sex, WHR, diet quality, physical activity category, and metabolic equivalents/day. There remains the potential for residual confounding due to unmeasured confounders, such as socioeconomic status, but their influence might be expected to be less than that of sex, diet, physical activity, and adiposity.

Our results demonstrated that lymphocyte count, monocyte-to-HDL-C ratio, neutrophil-to-HDL-C ratio, and platelet count, but not hsCRP level, SII, or NLR, were positively associated with BP, arterial stiffness, and HR in young adults. The stronger associations in males than in females may reflect the higher BP and variability in males or the greater potential for inflammation-associated diseases among males. While the effect sizes of some associations, such as the predicted marginal mean changes in SBP (e.g., ∼0.5–1.5 mmHg per one standard deviation increase in certain inflammatory markers, as depicted in Figure 2), may appear modest in absolute terms, their clinical significance in a young and healthy population warrants emphasis. Even small, chronic elevations in blood pressure or subtle increases in arterial stiffness from a young age can have profound cumulative effects over a lifetime, significantly increasing the risk of future hypertension and cardiovascular disease. Identifying these early, sub-clinical indicators is paramount for implementing timely preventive strategies. Unlike traditional markers that might become significant only in later stages of disease, these novel blood cell-derived indices, obtained from readily available and inexpensive routine blood tests, offer a practical avenue for early risk stratification. Their ability to predict subtle cardiovascular changes in healthy young adults could pave the way for proactive lifestyle interventions or targeted monitoring, potentially altering the long-term trajectory of cardiovascular health and reducing the burden of disease in adulthood.

## Data Availability

The data analyzed in this study is subject to the following licenses/restrictions: Data access is subject to restrictions imposed to protect participants' privacy. All researchers using Raine Study data must sign a data access agreement stipulating that data may not be released to anyone other than the investigators of the approved project. Additional details regarding data access are available at https://rainestudy.org.au/. Requests to access these datasets should be directed to https://rainestudy.org.au/.

## Supplementary material

Supplementary Figure S1 CONSORT Flow Diagram of Participant Inclusion and Data Availability from the Gen2 Raine Study Cohort. The flow diagram illustrates the selection process from the total Gen2 Raine Study cohort (*N* = 2,868) for inclusion in the current analysis. It details the number of participants eligible for follow-up, those who completed assessments at age 17 and age 22, and the availability of key data for clinical blood pressure (BP), inflammatory markers, and pulse wave analysis (PWA)/pulse wave velocity (PWV). The diagram further delineates the derivation of the final cohort (*N* = 693) used for longitudinal analysis of BP and inflammatory markers, and the cohorts for cross-sectional analysis of AIx (*N* = 645) and PWV (*N* = 666) at age 17. It also indicates the application of multiple imputation to create 20 datasets for statistical analysis. N, number of participants; BP, blood pressure; PWA, pulse wave analysis; PWV, pulse wave velocity; AIx, augmentation index; MI, multiple imputation.

Supplementary Figure S2 Scatter plot showing Spearman correlation between inflammatory markers and SBP.

Supplementary Figure S3 Scatter plot showing Spearman correlation between inflammatory markers and age.

Supplementary Figure S4 Scatter plot showing Spearman correlation between inflammatory markers and sex.

Supplementary Figure S5 Scatter plot showing Spearman correlation between inflammatory markers and waist-to-hip ratio.

Supplementary Figure S6 Scatter plot showing Spearman correlation between inflammatory markers and metabolic equivalents used per week.

Supplementary Figure S7 Scatter plot showing Spearman correlation between inflammatory markers and Western Diet factor score.

Supplementary Figure S8 Scatter plot showing Spearman correlation between inflammatory markers and smoking status.

Supplementary Figure S9 Predicted marginal mean effects of inflammatory markers on clinic heart rate for males (*N* = 363) and females (*N* = 330).

Supplementary Figure S10 Predicted marginal mean effects of inflammatory markers on clinic pulse pressure for males (*N* = 363) and females. (*N* = 330).

Supplementary Figure S11 Predicted marginal mean effects of inflammatory markers on pulse wave velocity for males (*N* = 349) and females. (*N* = 317).
