# Supplementary figures and images for: Associations of novel blood-derived markers of inflammation with blood pressure, arterial stiffness and heart rate in young adults
Source: Front Cardiovasc Med. 2025 Nov 25;12:1678178. doi: 10.3389/fcvm.2025.1678178 (PMC12685874; doi:10.3389/fcvm.2025.1678178)

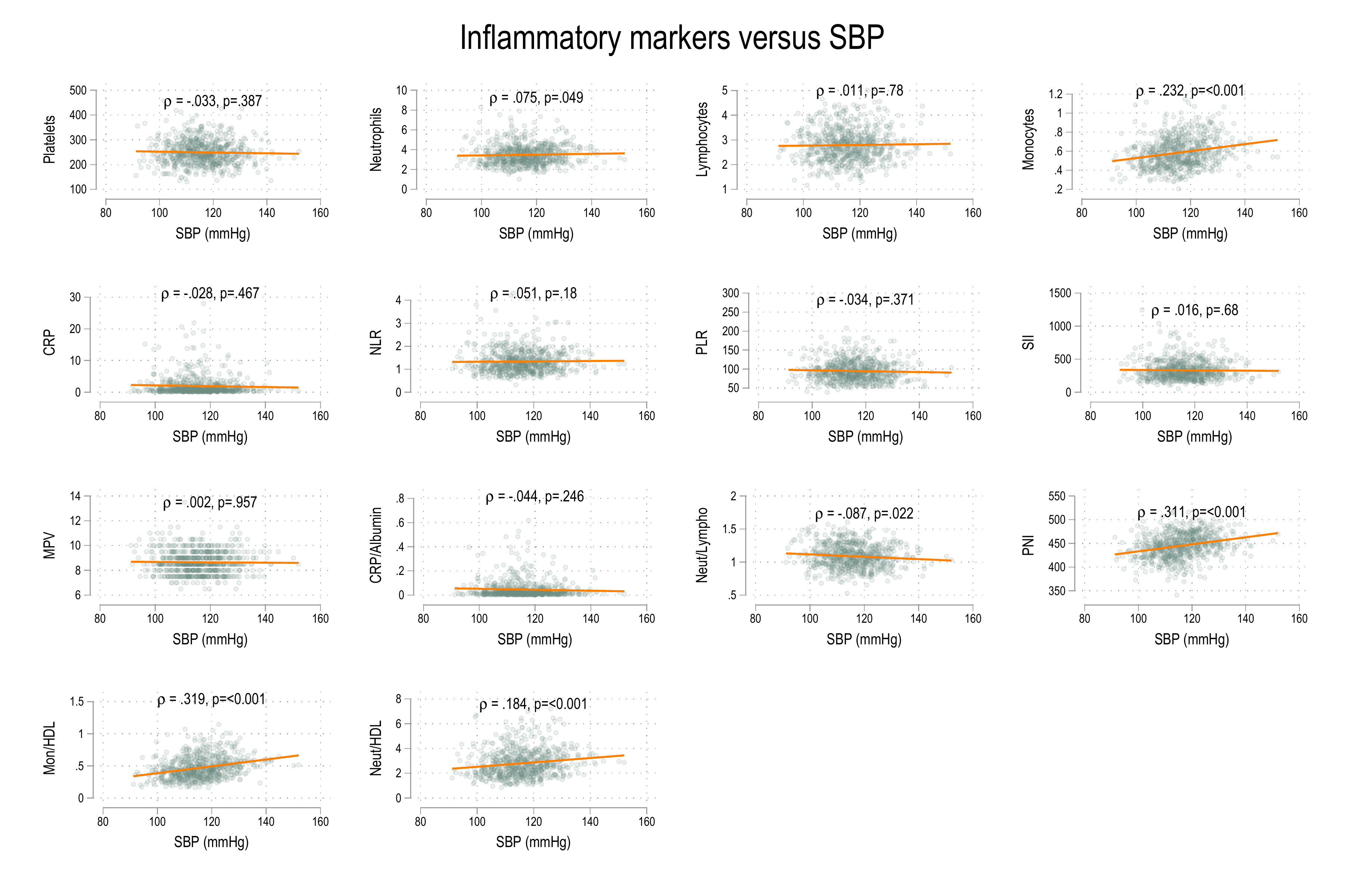

Supplement: Supplementary Figure S1 — CONSORT Flow Diagram of Participant Inclusion and Data Availability from the Gen2 Raine Study Cohort. The flow diagram illustrates the selection process from the total Gen2 Raine Study cohort (N = 2,868) for inclusion in the current analysis. It details the number of participants eligible for follow-up, those who completed assessments at age 17 and age 22, and the availability of key data for clinical blood pressure (BP), inflammatory markers, and pulse wave analysis (PWA)/pulse wave velocity (PWV). The diagram further delineates the derivation of the final cohort (N = 693) used for longitudinal analysis of BP and inflammatory markers, and the cohorts for cross-sectional analysis of AIx (N = 645) and PWV (N = 666) at age 17. It also indicates the application of multiple imputation to create 20 datasets for statistical analysis. N, number of participants; BP, blood pressure; PWA, pulse wave analysis; PWV, pulse wave velocity; AIx, augmentation index; MI, multiple imputation. [file Image1.tif]

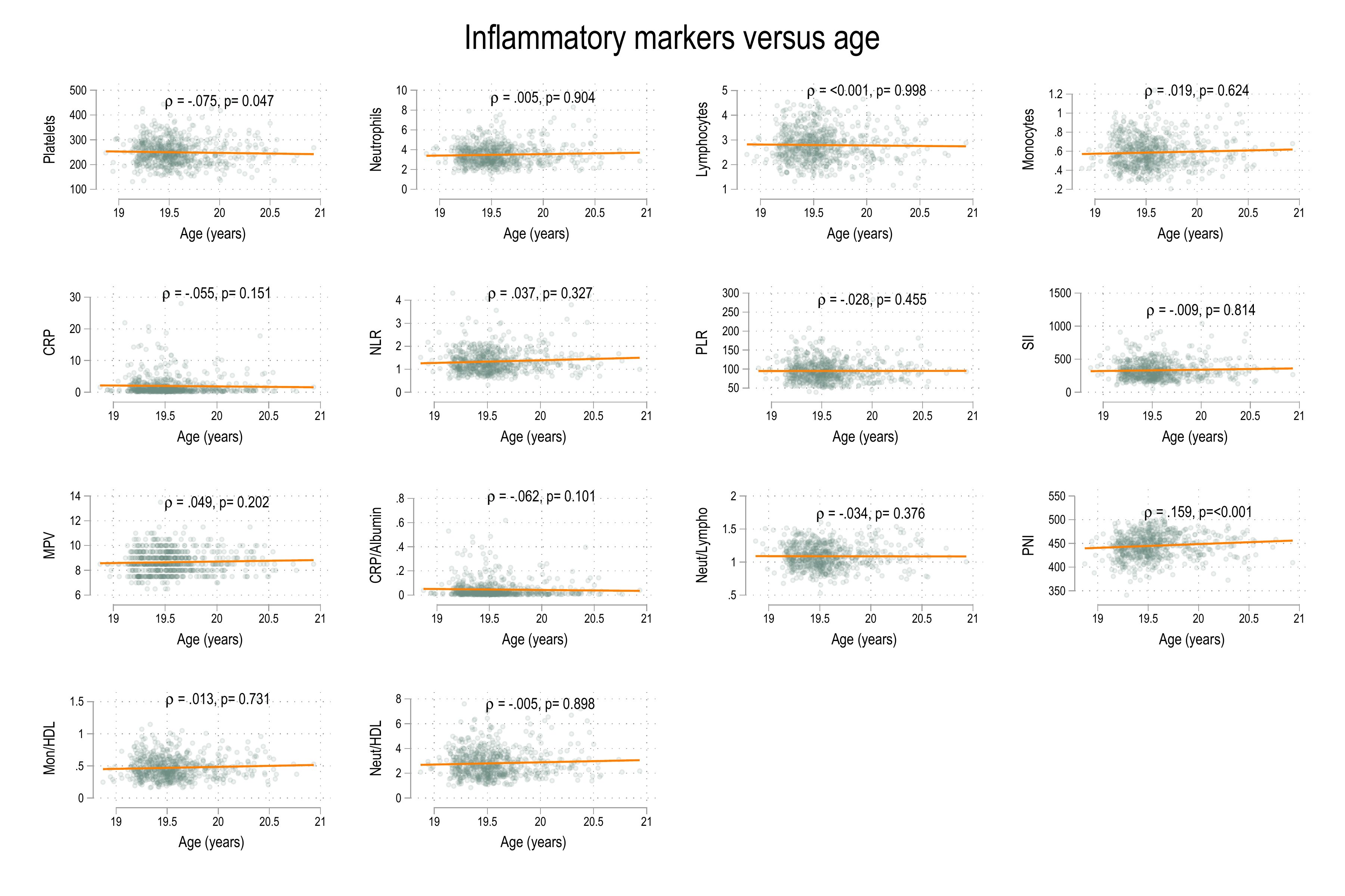

Supplement: Supplementary Figure S2 — Scatter plot showing Spearman correlation between inflammatory markers and SBP. [file Image2.tif]

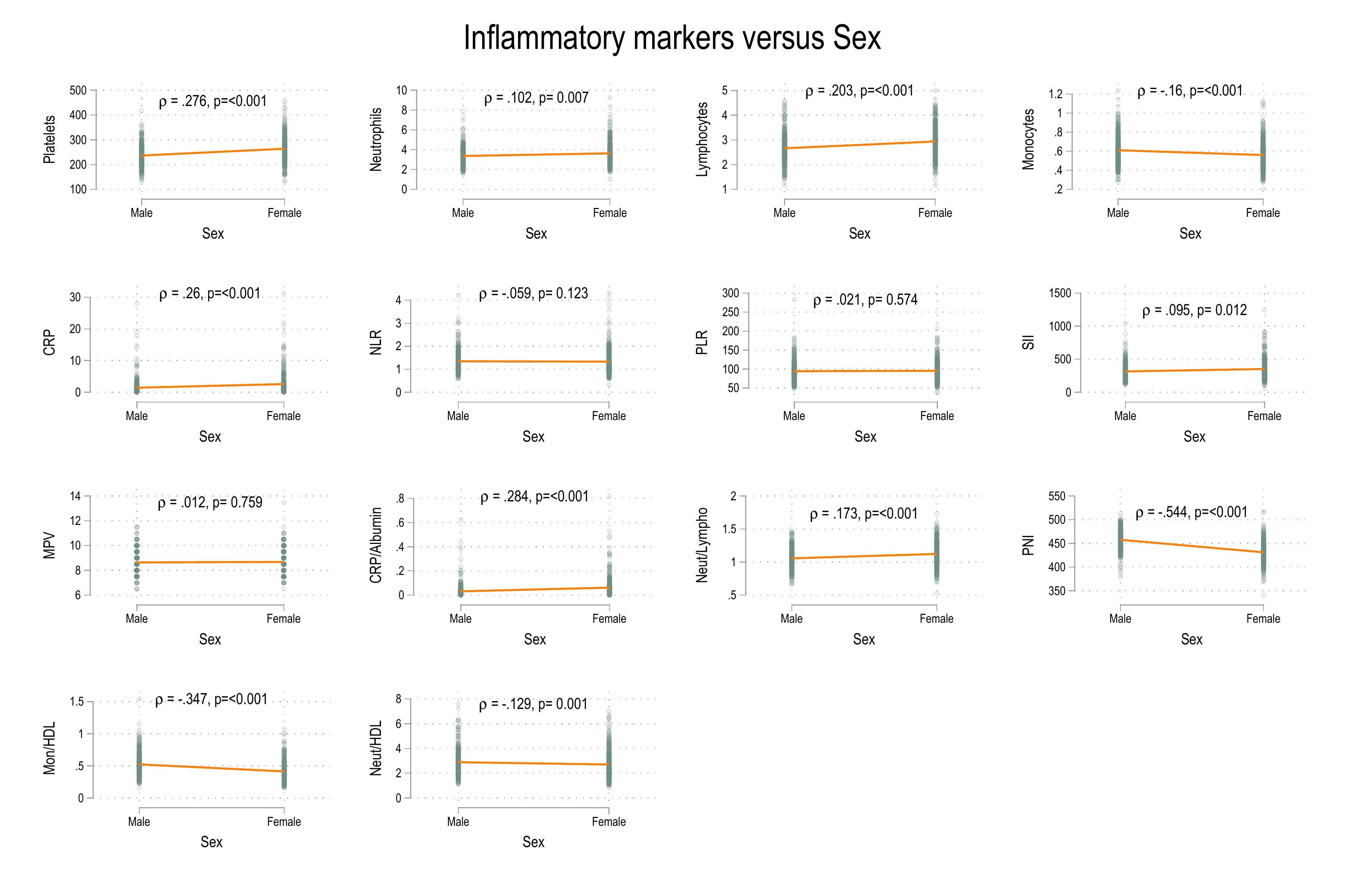

Supplement: Supplementary Figure S3 — Scatter plot showing Spearman correlation between inflammatory markers and age. [file Image3.tif]

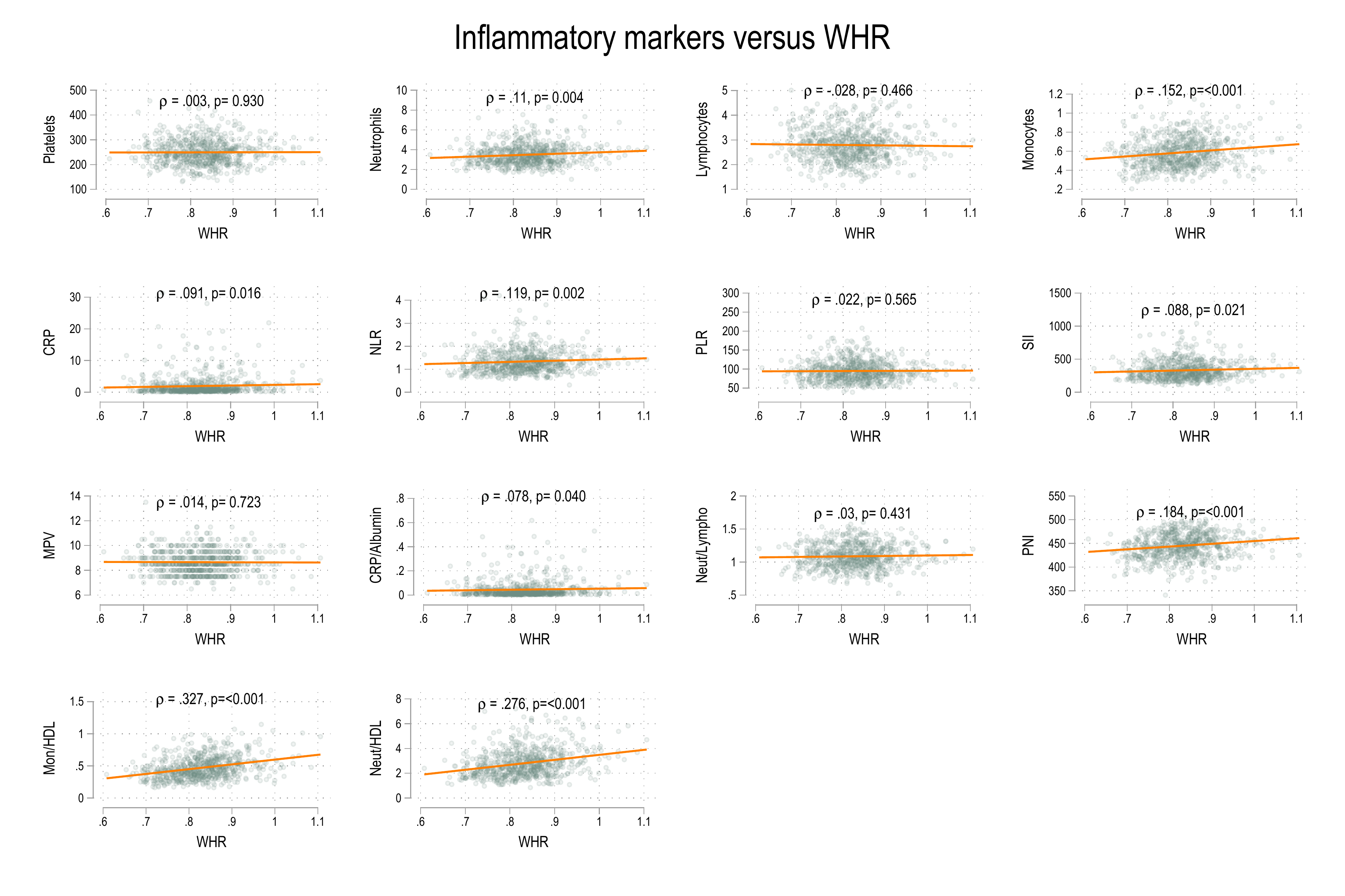

Supplement: Supplementary Figure S4 — Scatter plot showing Spearman correlation between inflammatory markers and sex. [file Image4.tif]

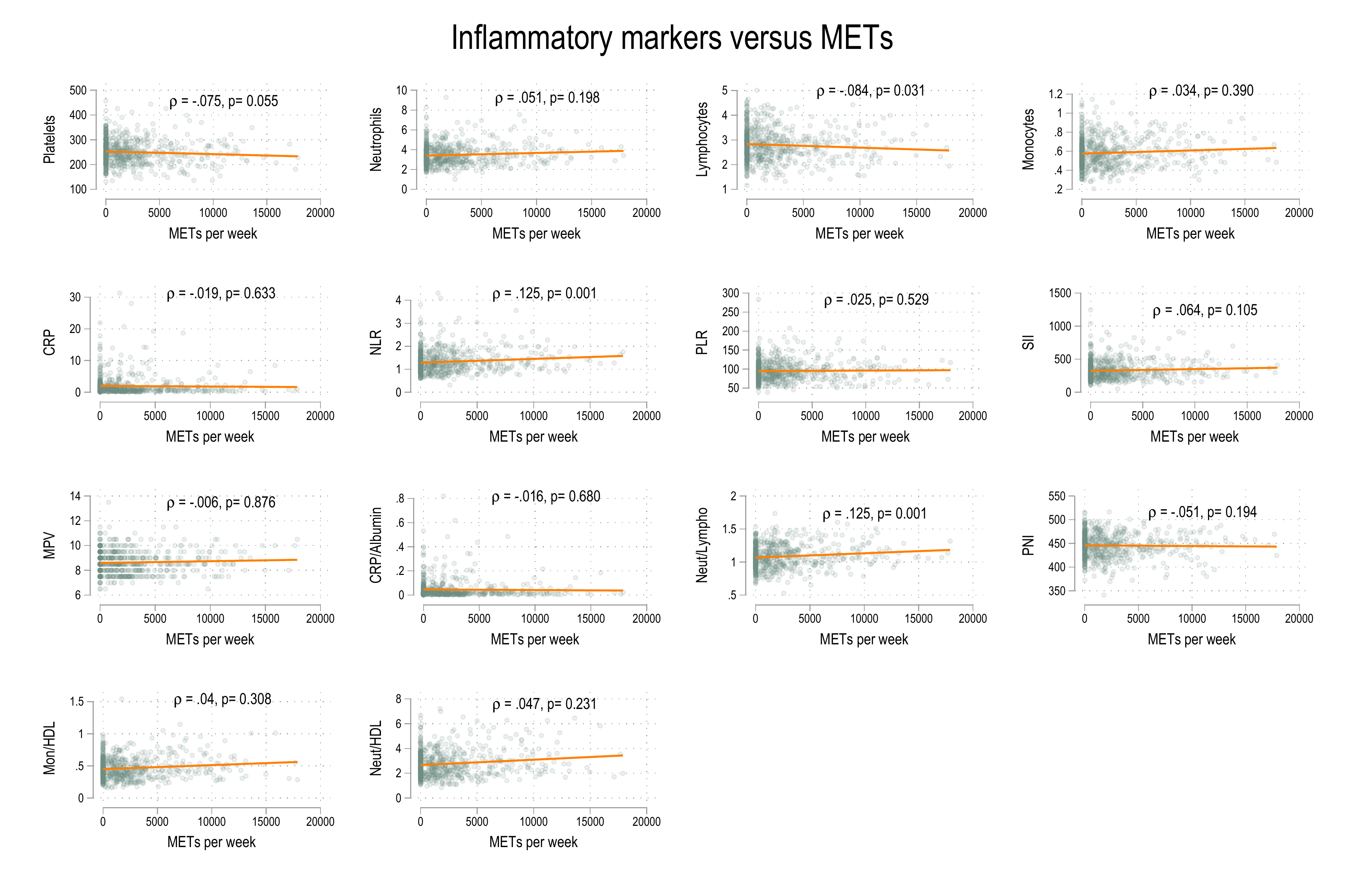

Supplement: Supplementary Figure S5 — Scatter plot showing Spearman correlation between inflammatory markers and waist-to-hip ratio. [file Image5.tif]

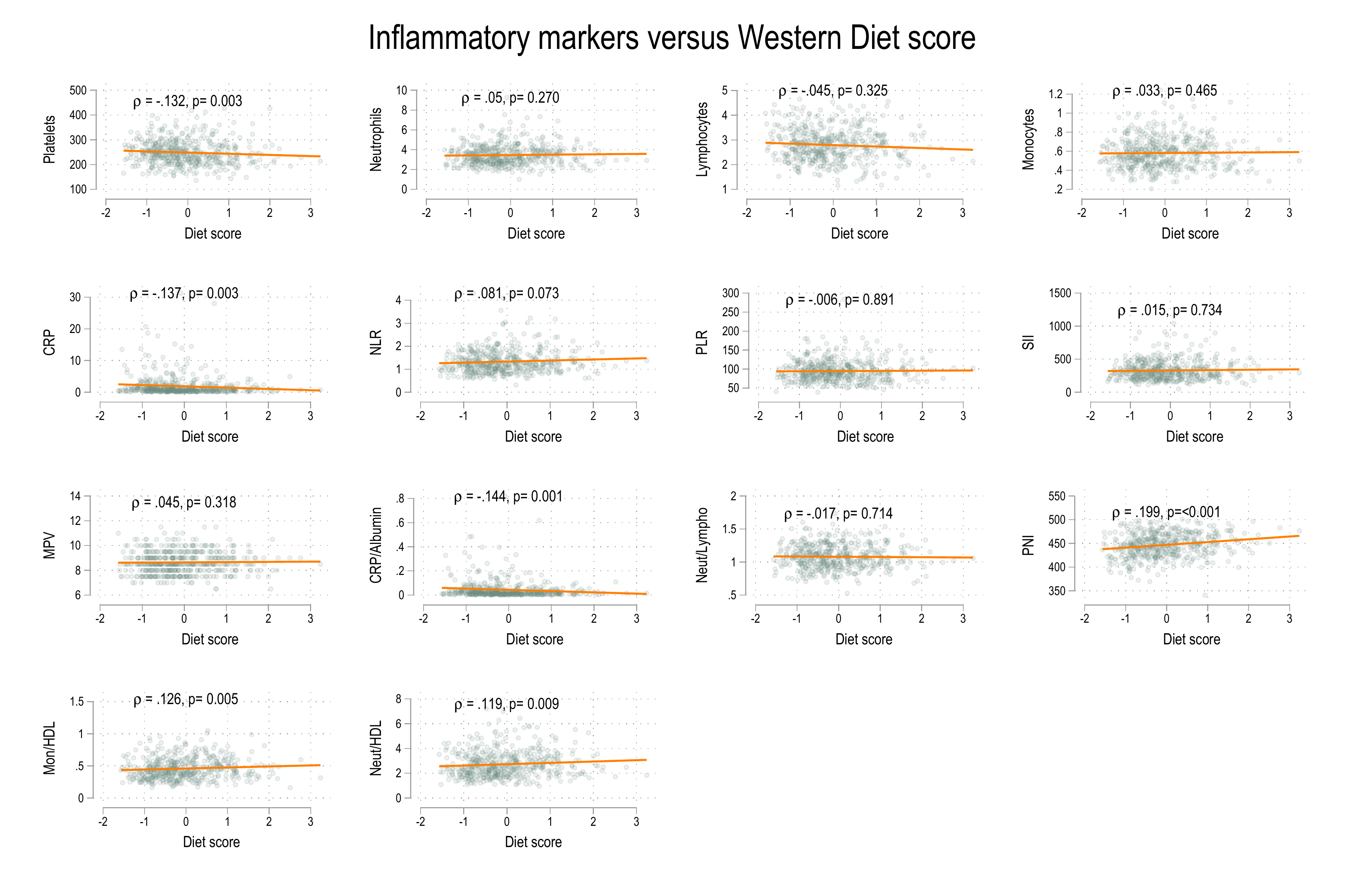

Supplement: Supplementary Figure S6 — Scatter plot showing Spearman correlation between inflammatory markers and metabolic equivalents used per week. [file Image6.tif]

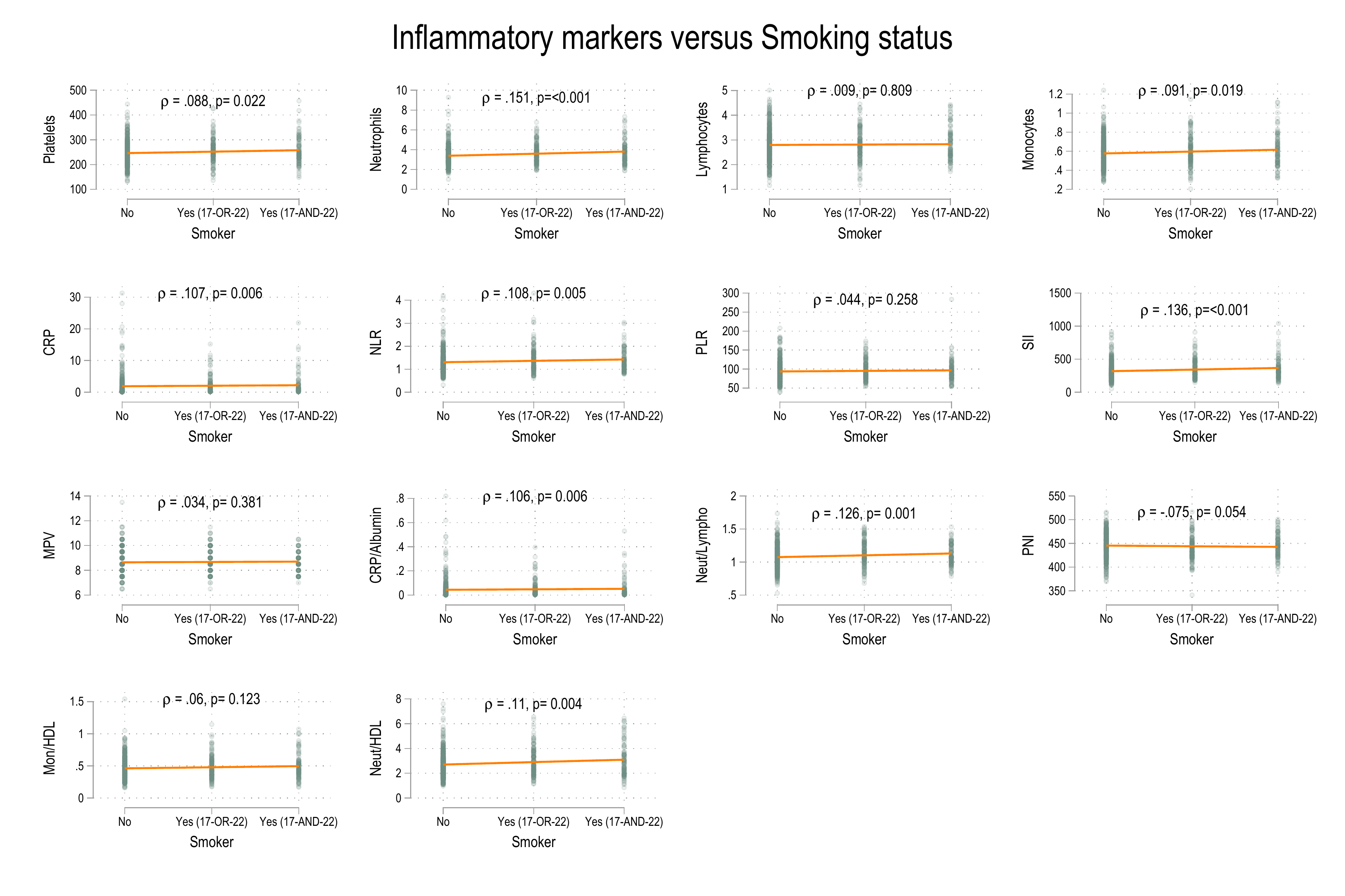

Supplement: Supplementary Figure S7 — Scatter plot showing Spearman correlation between inflammatory markers and Western Diet factor score. [file Image7.tif]

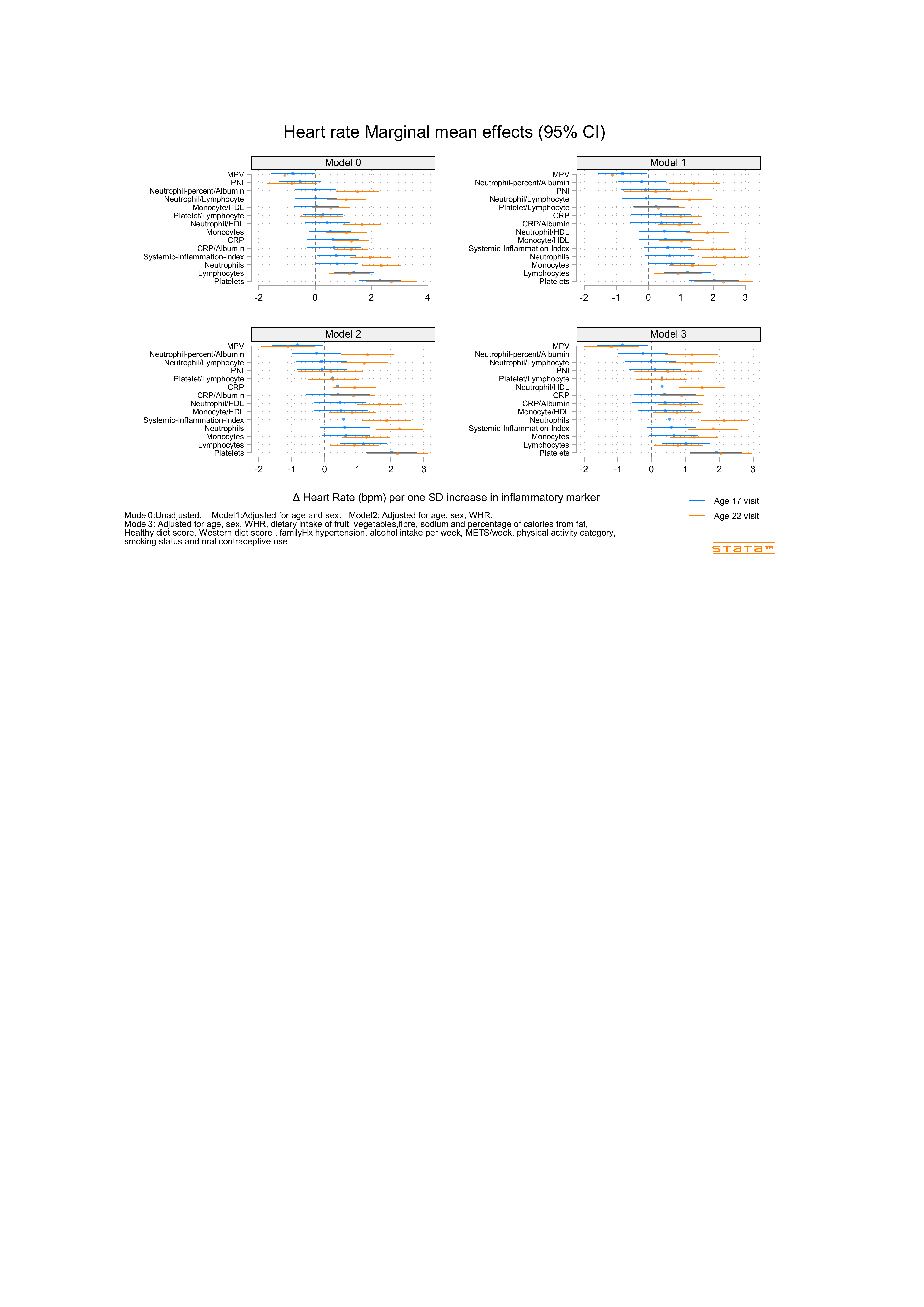

Supplement: Supplementary Figure S8 — Scatter plot showing Spearman correlation between inflammatory markers and smoking status. [file Image8.tif]

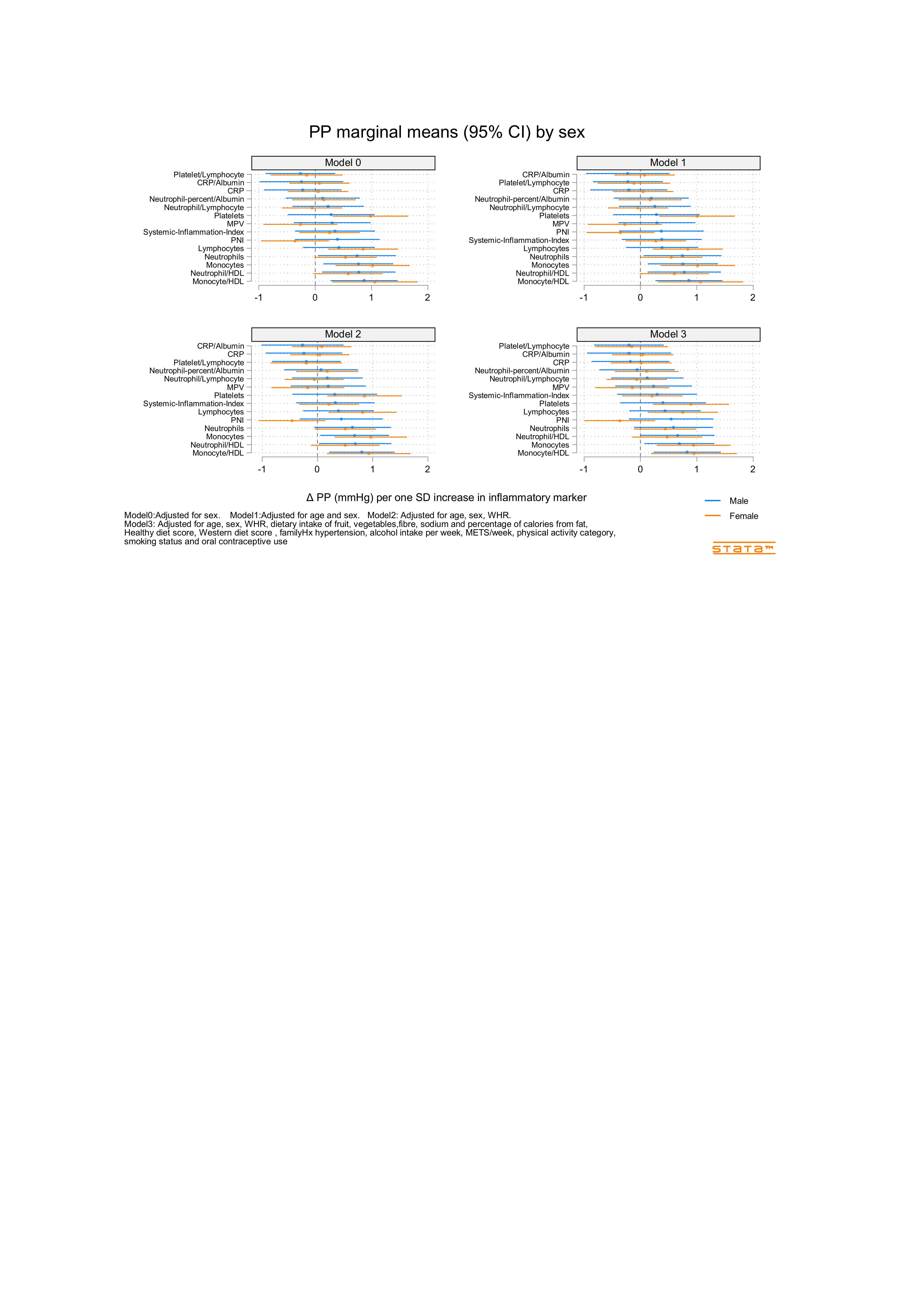

Supplement: Supplementary Figure S9 — Predicted marginal mean effects of inflammatory markers on clinic heart rate for males (N = 363) and females (N = 330). [file Image9.tif]

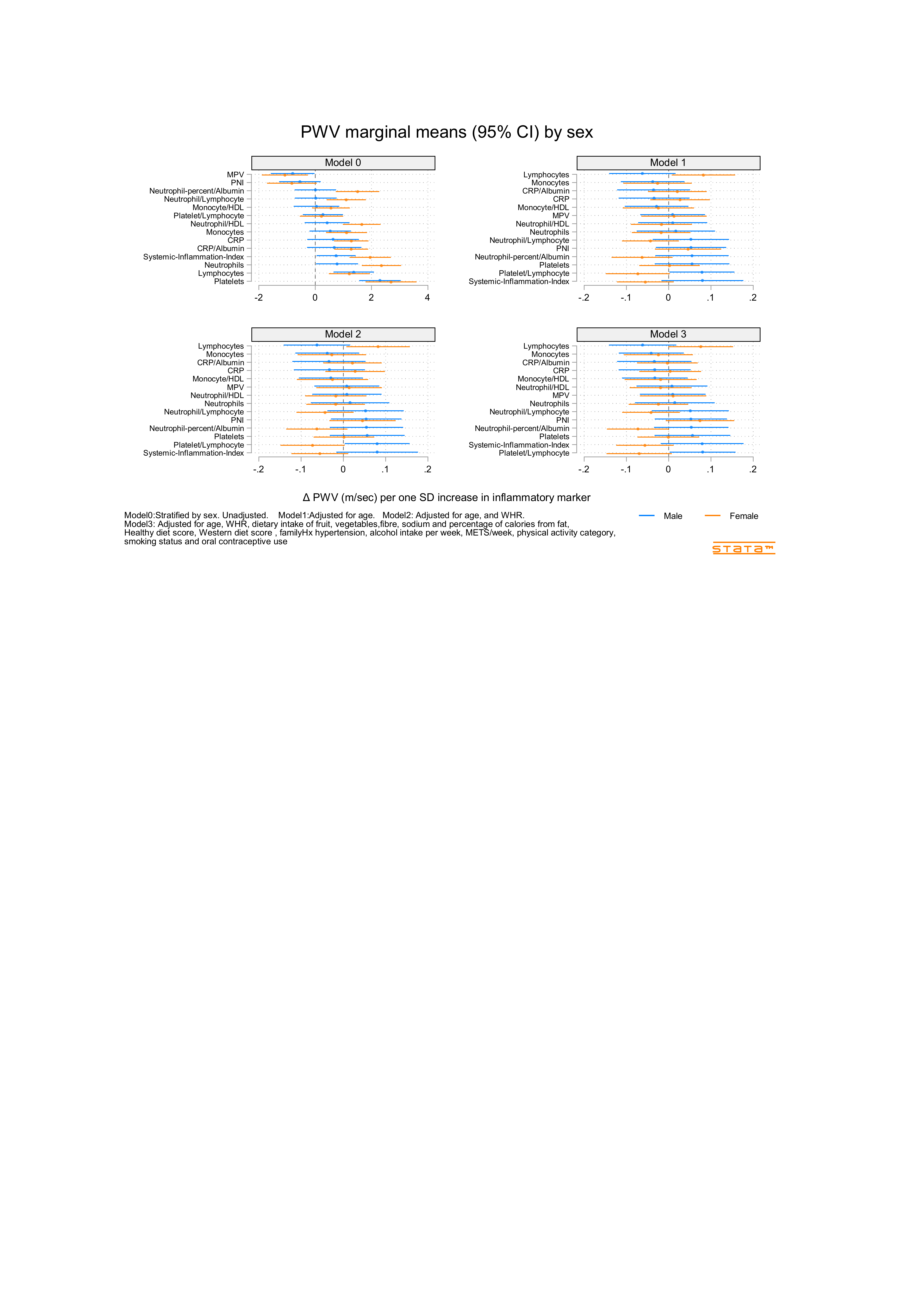

Supplement: Supplementary Figure S10 — Predicted marginal mean effects of inflammatory markers on clinic pulse pressure for males (N = 363) and females. (N = 330). [file Image10.tif]

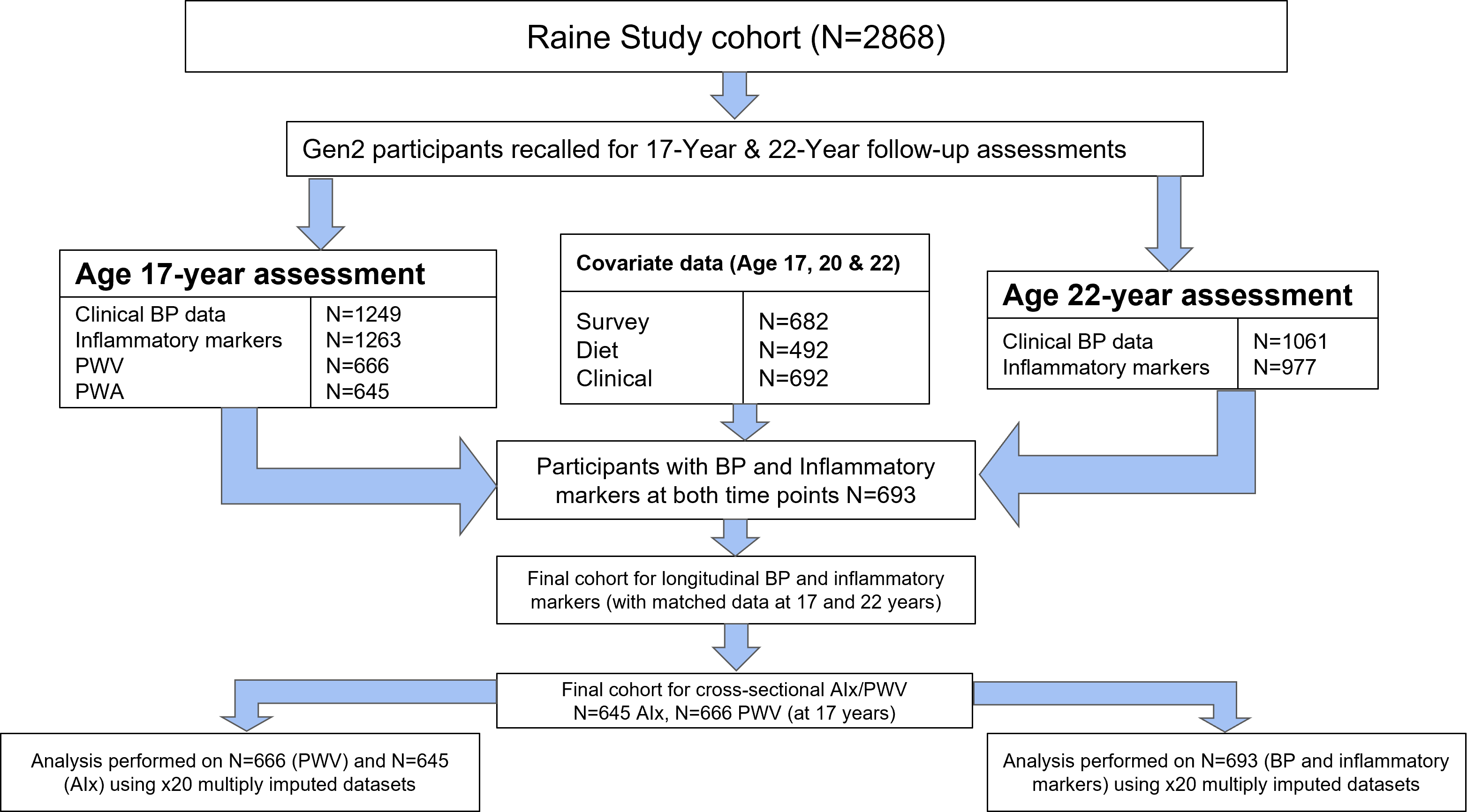

Supplement: Supplementary Figure S11 — Predicted marginal mean effects of inflammatory markers on pulse wave velocity for males (N = 349) and females. (N = 317). [file Image11.tif]
